# Classification of land lot shapes in real estate sector using a convolutional neural network

**DOI:** 10.1371/journal.pone.0308788

**Published:** 2024-09-19

**Authors:** Subin Ham, Changro Lee

**Affiliations:** Department of Real Estate, Kangwon National University, Chuncheon, Gangwon-do, Republic of Korea; Manipal Institute of Technology, INDIA

## Abstract

In the agriculture and real estate industries, land lot shapes have mostly been classified by visual inspection or hard-crafted rules. These conventional methods are time-consuming, resource-intensive, and subject to human bias. This study aims to fill this gap and alleviate problems inherent in traditional lot classification approaches. This study attempts to classify lot shapes automatically, using a convolutional neural network. A study area was chosen, image data of the lots in the area were collected and preprocessed, and an Xception neural network was specified to classify land lots according to their shapes. The test applied to a different area adjacent to the study area achieved an accuracy of 90.1% and area under the curve (AUC) of 0.96. Additionally, this study demonstrated that shape regularity can be quantified using the output scores from the neural network analysis. This is the first attempt to employ a deep learning algorithm for land management on a micro-spatial scale. The classification approach proposed in this study is expected to encourage the rapid and accurate classification of various lot shapes.

## 1. Introduction

Lot shapes have been analyzed across diverse industries such as farming, urban planning, and property valuation. In many countries, agricultural policymakers aim to consolidate fragmented land lots to enhance productivity; thus, understanding lot shapes is essential for implementing land consolidation programs. In urban planning, the shapes of land lots play a crucial role in designing street layouts and optimizing zone regulations. In property valuation, the shape of a lot can significantly impact its market value because regular shapes, such as squares, are often easier to develop and therefore command higher prices. Studies on land lot shapes have aided in improving agricultural productivity, increasing social benefits generated from urban development projects, and enhancing property appraisal accuracies.

Despite the importance of lot shape classification, many shapes are still classified manually. That is, land lots have been classified through visual inspection by human observers or by using hard-crafted rules. Common rules observed in previous studies include the ratio of depth to frontage in a lot, simplicity index, and roundness index [1–3]. However, visual inspection is time-consuming, resource-intensive, and subject to human bias. With hard-crafted rules, the proposed indices are often arbitrary and suitable only for the selected study areas they were designed for [4].

This study aims to automate land lot classification without human involvement and simultaneously increase classification accuracy. Specifically, we attempt to classify land lots according to their shapes using a deep learning algorithm. A study area consisting of approximately 20,000 lots was chosen, and the lots were automatically classified using a convolutional neural network. Convolutional neural networks, which are specialized image recognition tools, are widely used across various domains, including the analysis of medical images for diagnoses such as breast cancer [5] and facial recognition for authentication [6]. We also attempt to quantify shape regularity by utilizing the output scores obtained from the neural network adopted in this study. We demonstrate that a deep learning-supported approach to land-shape classification can replace existing methods effectively and efficiently.

In this study, a relevant neural network was trained by feeding imagery data to the network to classify lot shapes. This approach has the advantage of eliminating human involvement, enabling lot shapes to be classified accurately and promptly. Although neural networks specialized in computer vision have been extensively used in related fields, such as landform classification [7, 8] and plant classification [9], this technique has not been applied to the pattern analysis of land lots. To the best of our knowledge, this study is the first to employ a deep learning algorithm for land management on a micro-spatial scale.

The remainder of this paper is organized as follows. Section 2 reviews prior studies on lot shapes in rural and urban areas, and the emerging trend of image data and neural networks usage. Section 3 explains the dataset, study area, and neural network architecture. The classification results and discussion are presented in section 4. Finally, the findings are summarized and a path for future research is suggested in section 5.

## 2. Literature review

### 2.1 Classification of lot shapes in rural and urban areas

Studies on effective lot shapes started in the agricultural industry. In his seminal paper, Losch [10] proposed that a hexagonal lot is the most efficient for farming operations. Lee and Sallee [11] argued that a rectangular shape is superior to a hexagonal one when considering the costs of farming operations. Since then, there have been a few discussions about which shape is more productive for farming [12, 13]; the rectangle-shaped lot is accepted as a standard lot shape in farming practice today. These studies focused on large lots in rural regions and primarily aimed to minimize tillage time per hectare in land consolidation [14]. The lot shape was manually classified into a few groups after visually examining sample lots in the study area, such as rectangular, triangular, trapezoidal, and irregular shapes [15].

Later, lot shapes in cities were actively investigated following early studies on farmlands. In urban planning, the lot and its shape are the basic units for block subdivision, and different lot patterns are usually suggested for different block types [16]. Lot shape is also considered a critical determinant for urban redevelopment, and Gao and Asami [1] examined the impact of lot shape on the costs and benefits of redevelopment projects. They concluded that irregularly shaped lots provide landowners with fewer benefits, that is, higher reconstruction costs and lower price escalations between the original and redeveloped lots. In addition, lot shape plays an important role in property valuation: rectangular lots are typically appraised at higher prices than irregularly shaped ones because improvements can be made more efficiently on the former [17]. These studies focused on small-sized lots in urban areas and attempted to maximize the social benefits generated from urban planning and redevelopment, or enhance the accuracy of property valuation. Similar to studies in the agricultural industry, lot shape was categorized in a qualitative manner such as regular, intermediate, and irregular [18].

Recently, geographical information system (GIS)-driven classification methods have been proposed [2, 4, 19]. These studies used several GIS algorithms to calculate shape-perception indicators, such as simplicity index and roundness index, and compared different urban configurations, including planned, organic, tree-like, and grid-like layouts.

Conventional methods for classifying lot shapes, such as computing shape-perception indicators, have two main advantages. First, indicators, such as the roundness index, are straightforward and intuitive. Secondly, the calculation of these indices typically involves simple formulas and algorithms, thereby making them computationally efficient.

In short, previous studies employed two main approaches: visual inspection by human observers and the use of hard-crafted rules. The visual inspection approach is significantly hindered by the inspector’s subjectivity. For example, a trapezoidal land lot may be classified as irregular or rectangular depending on the inspector’s perspective. The vague definition of the lot shape also contributes to this subjectivity. The latter approach, which relies on hard-crafted rules, is generally applicable to relatively regular-shaped lots. Previous studies using this approach primarily analyzed rectangular lots and their variants for simplicity. Their main index was to calculate the depth-to-frontage ratios or similar metrics of typical rectangular lots and compare them with the measurements of other lots. Although more sophisticated indices, such as the compactness metric, were developed later [20, 21], this approach is still limited because it can be practically applied only to rectangular or quasi-rectangular lots.

We attempt to train a deep learning model by feeding lot-shape data into the model, to enable lot-shape recognition and classification without human judgement. This approach offers several advantages and fills a research gap. First, this study addresses the entire range of lot shapes found in practice, from highly irregular to perfectly square lots, offering a more inclusive and holistic approach for lot shape classification. Second, this study introduces the use of deep learning models for lot shape recognition and classification. By training these models with large amounts of shape data, this study moves away from manually crafted rules and human inspections. Finally, by leveraging advanced deep learning techniques, this study enhances the accuracy of lot shape classification.

### 2.2 Utilization of image data and neural networks

After lot shapes in cities were rigorously analyzed, studies on lot patterns were phased out from the literature for a while. Around 2015, research on shape was revived following the rising popularity of deep learning. Deep learning technique has spread quickly in all industries within a short time, and the neural network has emerged as the de facto standard model to implement this technique. Neural networks have excellent capabilities that cannot be realized through conventional models like regression analysis. These capabilities include capturing non-linear relationships in data, transferring knowledge learned in one task to another, and handling unstructured data [22]. In particular, the ability to process unstructured data, such as images and sounds, enables neural networks to achieve excellent performance in computer vision and speech recognition [23–26].

In tandem with these trends, land shapes were analyzed through different approaches compared to prior studies: using neural networks and imagery data. Sunaga et al. [7] collected satellite images, fed them into convolutional neural networks, and classified similarly shaped terrains, such as plains, ridges, and volcanoes. The classification of land forms by exploiting neural networks and satellite images has become a popular research topic in the literature [8, 27, 28]. Neural networks and visual data have also been used in the building architecture industry to classify floor plans. Ahn [29] collected floor plan images and analyzed the shapes of apartment units using convolutional neural networks. Like studies on land form classification, the investigation of geometric elements in property floor plans within the framework of deep learning is a recent and popular research theme in building design [30–32].

Although neural networks and images have been actively exploited to classify land forms and floor plans, this approach has not yet been applied to the analysis of lot shapes. To the best of our knowledge, this study is the first attempt to classify lot shapes on a microscale using a deep learning algorithm: lot shapes are not manually classified by visual inspection, but through a learning process based on image data. By adopting this approach, we demonstrate that lots can be classified more efficiently, and without human bias.

## 3. Dataset and architecture of a convolutional neural network

### 3.1 Dataset and study area

Peongsan-gu was chosen as the study area. It is a district in Changwon city, South Korea. Seongsan-gu is characterized by a mixed landscape of downtown urban and surrounding rural areas. Thus, it contains various lot shapes, from square-shaped lots in the downtown areas to extremely irregular-shaped lots in the suburbs.

**Fig 1:**
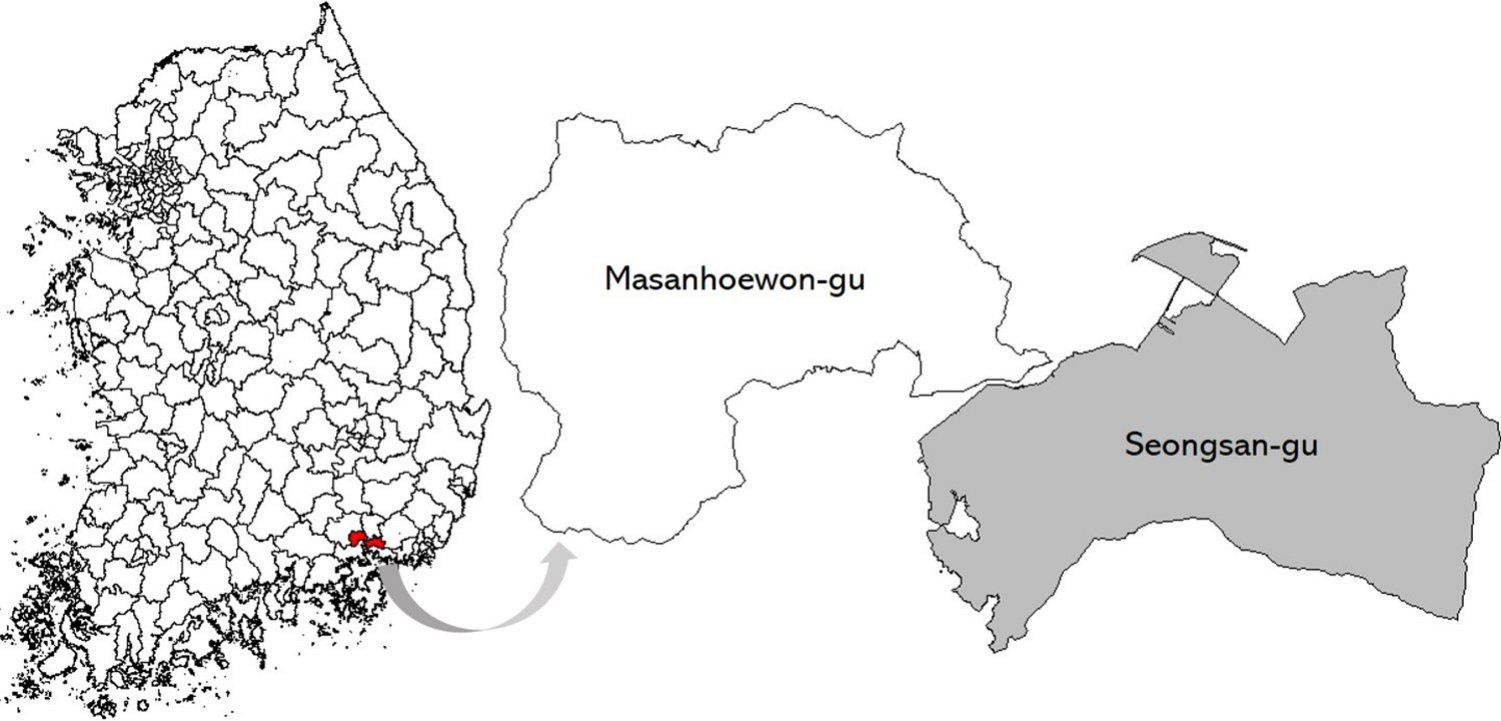
Study and test areas (Figure created by the authors).

Approximately 20,000 lots comprise Seongsan-gu, and image files (JPG format) for 18,976 lots were collected. Of these lots, 8,309 (44%) were labeled as regular shapes, and 10,667 (56%) were labeled as irregular shapes. Tax assessors determine shapes by visually examining lots on the cadastral map. Thus, these data can be considered balanced in terms of the distribution of labels. The 18,976 lots were further randomly split into 80% training and 20% validation data. Finally, the test dataset was obtained from a different area to reliably ascertain model validity. A part of lots in Masanhoewon-gu (3,845 lots) were used for testing. Masanhoewon-gu is another district in Changwon city which is bordered by the study area (Seongsan-gu) to the northwest. Table 1 presents the dataset configuration and Fig 1 shows the study and test areas.

**Table 1:** Dataset configuration.

Label		Training data (80%)	Validation data (20%)	Test data
Regular	8,309	6,647	1,662	1,497
Irregular	10,667	8,534	2,133	2,348
Total	18,976	15,181	3,795	3,845

**Fig 2:**
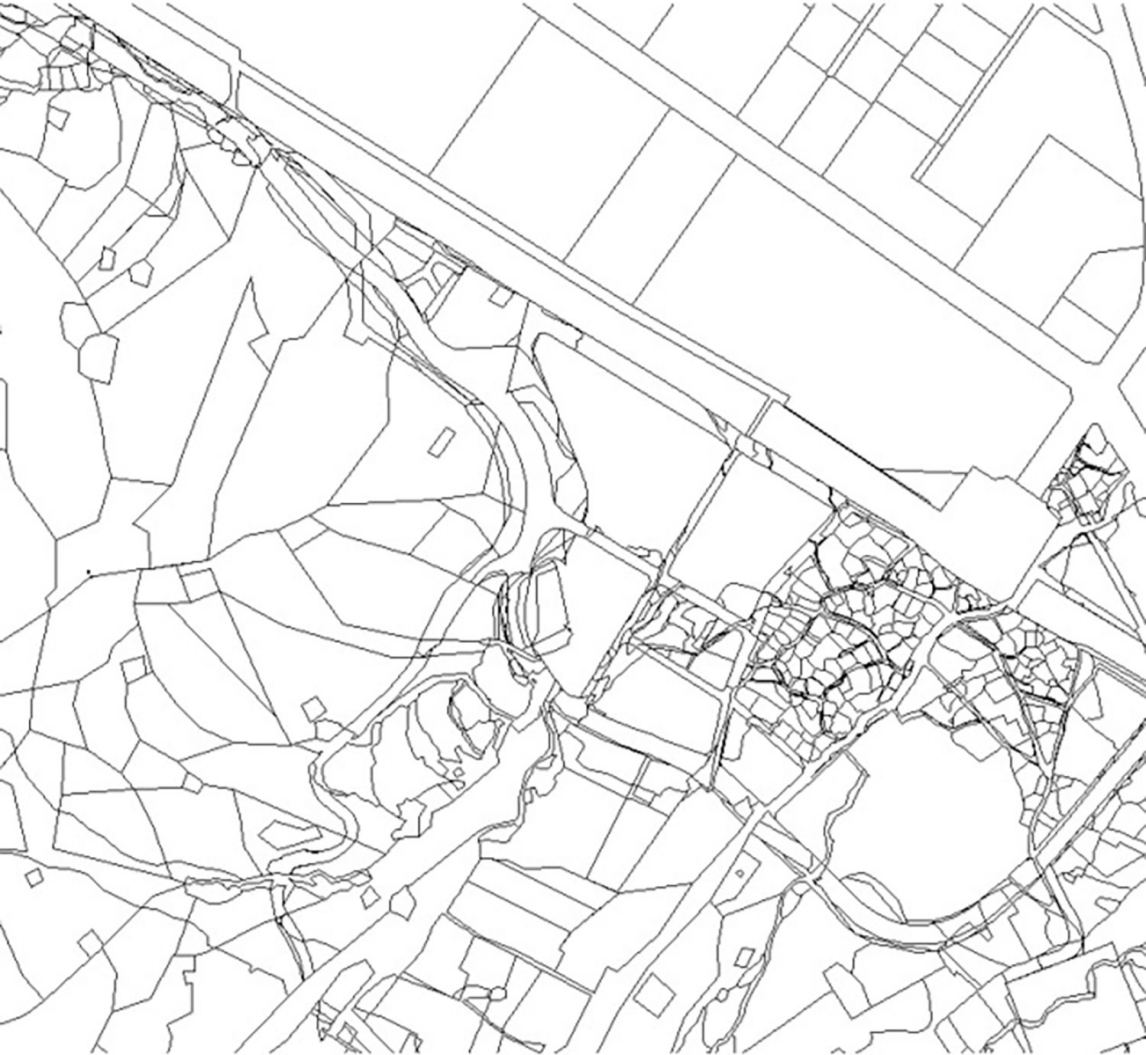
Study area at a lot-level resolution (Figure created by the authors).

Fig 2 shows a part of the study area at lot-level resolution. The northern area is characterized by rectangular lots, which are typical landscapes of downtown areas. In contrast, the southern area consists mainly of irregularly shaped lots, which are frequently observed in rural areas. This study attempts to classify lots with a wide spectrum of shape regularities, as shown in Fig 2.

### 3.2 Specifying a neural network

This study adopted the architecture of an Xception neural network. The Xception neural network is one of the convolutional neural networks, and has the advantage of using network parameters more efficiently compared to other convolutional neural networks [33].

**Fig 3:**
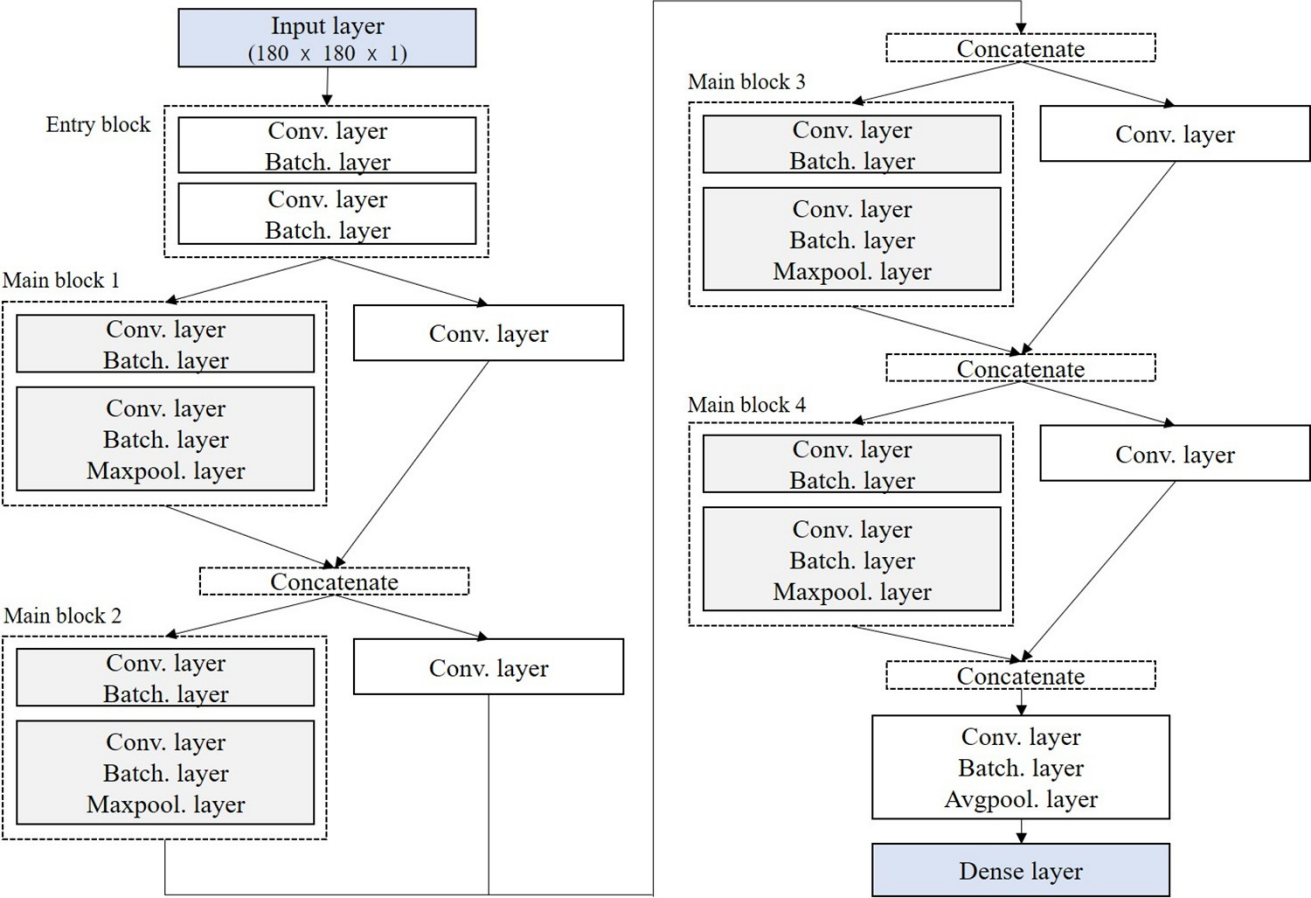
Architecture of the neural network. Notes: Conv. layer, Batch. layer, Maxpool. layer, and Avgpool. layer represent the convolutional, batch normalization, maximum pooling, and average pooling layers, respectively.

Lot images were prepared in a 180×180 pixel-size and standardized to have values between zero and one. Data augmentation was applied to the training data. That is, horizontal flipping and random rotation techniques were used. This augmentation procedure helps the neural network learn different aspects of the images. The neural network architecture consists of one entry block and four repeated main blocks. The convolutional and pooling layers primarily comprise these blocks. At the end of the architecture, a final classification layer (an ordinary dense layer) was added to classify the land lots as regular or irregular. Fig 3 shows the network architecture used in this study. The architecture presented in the figure was determined by evaluating its performance on validation data during training. This architecture is well-suited for the dataset in this study for several reasons. First, it performs spatial convolutions on each channel of the input separately before mixing outputs via pointwise convolutions. This reduces the required number of parameters compared with traditional convolutional layers, thereby enhancing the network efficiency without sacrificing performance. Second, it is computationally efficient and tends to outperform networks of similar sizes, making it suitable for implementation in environments with limited computational resources, such as personal computers [33].

The neural network processes input images as follows. First, it starts with an image of a predefined size (180×180 pixels). The entry block extracts high-level features from the image. The main blocks further process the features extracted by the entry block. These blocks are composed exclusively of depth-wise separable convolutions. The exit block prepares the feature maps for classification and passes the output to a dense layer. The final dense layer uses a sigmoid activation function for classification. During training, the neural network uses backpropagation to compute the gradient of a binary cross-entry loss function. An adaptive moment estimation (Adam) optimizer [34] with a learning rate of 0.001 was employed. A rectified linear unit (ReLU) activation function was utilized for all layers except for the last dense layer, where a sigmoid activation function was employed. The network was trained for 15 epochs with a batch size of 32.

## 4. Results

### 4.1 Classification accuracy

Land lots (3,845 samples) in the test area of Masanhoewon-gu were used to evaluate the performance of the neural network, and a threshold of 0.5 was adopted. Table 2 shows the result of the classification, in which approximately 90.1% of the test lots were classified correctly ((2,154+1,309)/3,845). A receiver operating characteristic (ROC) curve is another popular metric. The area under the ROC curve (AUC) was computed as 0.96. ROC and AUC metrics are especially relevant for datasets where target variables are imbalanced [35]. An AUC value of 0.5 indicates that the neural network has no discrimination capacity, while an AUC value of 1.0 indicates an ideal neural network that correctly classifies all classes.

**Table 2:** Result of the classification.

(N = 3,845)	Observed: Irregular	Observed: Regular
Predicted: Irregular	2,154	188
Predicted: Regular	194	1,309
Evaluation	Accuracy	AUC
	90.1%	0.96

A classification accuracy of 90.1% and an AUC of 0.96 can indicate good or poor performance depending on the task characteristics at hand. In some applications, achieving more than 50% accuracy may be sufficient, whereas even 99% prediction accuracy can be trivial for an extremely imbalanced classification problem. In the real estate sector, Chen et al. [36] achieved accuracies of 77–90% and AUC values of 0.75–0.93, respectively, for house image classification. In the healthcare industry, where convolutional neural networks are extensively applied, Li et al. [37] reported accuracies of 85–94% and AUC values of 0.84–0.94 for skin cancer and retinopathy image classifications.

In this study, we concluded that an accuracy of 90.1% and AUC of 0.96 are practically sufficient, especially given the absence of human involvement in the classification process. When comparing this accuracy and AUC value with those of previous studies, the results of this study are both comparable and effective. In addition, an AUC value close to 1.0 does not always imply a perfect neural network; it may indicate overfitting to the training data. Therefore, we believe that the neural network proposed in this study is suitable for industrial deployment with minimal modifications. Based on the accuracy metric, the remaining 9.9% of the misclassified land lots can be addressed separately by experts, such as assessors.

### 4.2 Quantifying shape regularity

**Fig 4:**
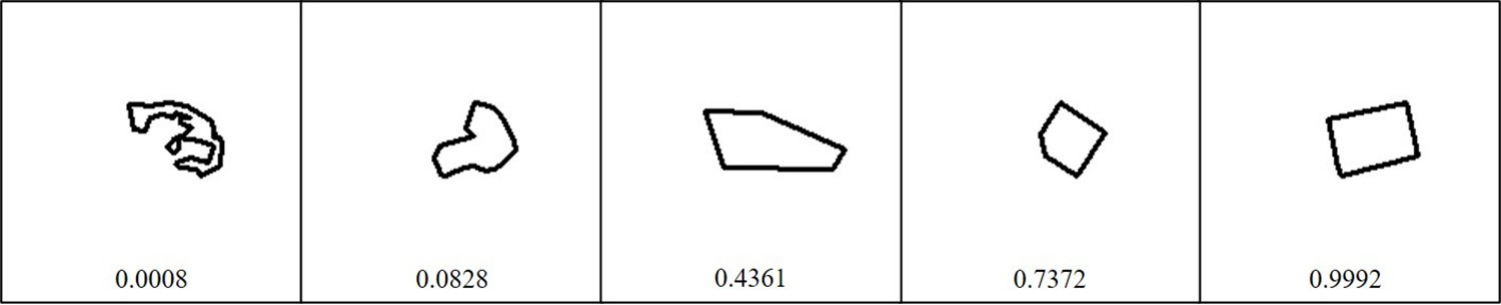
Land lots and probabilities of being regular-shaped lots.

In data preprocessing, regular-shape lots were coded as one, and irregular-shape lots were coded as zero. Thus, the output score from the binary classification neural network indicates the probability for a lot to be classified as regular. Fig 4 shows example lots with varying shapes and the corresponding probabilities of being regular-shaped lots.

The first lot in the figure received a probability of 0.0008, and is indeed extremely irregular-shaped; it can be easily inferred that this lot has a significant disadvantage during building, because its irregular shape may considerably reduce the effective area. Progressing from left to right, the lot shape approaches a square pattern. The far-right lot in the figure receives a probability of 0.9992, and it can be easily understood that its effective area for construction is almost equal to the original lot size. It is well-known that a land lot with a square pattern is preferred in the market and generally commands the highest price when other site characteristics are equal.

Lot shape is used as a price determinant in tax assessments for property in South Korea. However, the classification is simplified into five categories: flag-shaped, irregular, trapezoidal, rectangular, and square-shaped. Accordingly, the corresponding price discounts in the assessment are also limited to five values: 11%, 6%, 3%, 1%, and 0% [38]. These discount values are only valid in the study area: they differ depending on administration jurisdictions. Thus, the approach proposed in this study provides several advantages over the current assessment system. First, it can significantly reduce the subjectivity in human judgement on lot shape: it is well-known that the same lot can be classified differently by different people. Second, unlike the time-consuming visual inspection of lot shapes by humans, it can speed up the classification task without compromising classification quality. Finally, it can expand the current number of shape categories (five categories) and incorporate more diverse shapes: the probability of being a regular-shaped lot can be used as a criterion for such an expanded classification.

## 5. Conclusion

For long, lot shapes have been investigated in fields ranging from agriculture to urban planning and property valuation. However, these studies tended to focus on rectangular or quasi-rectangular lots while classifying them manually. Thus, this study attempted to classify land lots according to the various shapes observed in practice, and a classification was performed using image data and a neural network.

Seongsan-gu was chosen as the study area, 18,976 lots in the area were fed into a convolutional neural network, and the network was trained to classify lot shapes. Its performance was evaluated based on a test dataset from an area close to the study area, and the network achieved an accuracy of 90.1%. Finally, we demonstrated that the shape regularity of the lots can be quantified effectively using the output probability from the network. The approach proposed in this study is expected to be applicable to relevant industries, including property valuation.

This study employed a single-input and single-output neural network: an image file was fed into the network and a binary classification result was obtained. However, one superior capability of a neural network is its flexible design. For example, a multi-input and single-output neural network can be envisioned: an image file and metadata (lot area, zoning, frontage width) can be fed into a neural network simultaneously, and the price of the subject lot can be estimated as an output. In future research, a neural network should be designed to have a more diverse architecture so that it can be utilized in more extensive areas.
